# The Multifaceted Mechanism of Leptin Signaling within Tumor Microenvironment in Driving Breast Cancer Growth and Progression

**DOI:** 10.3389/fonc.2014.00340

**Published:** 2014-11-26

**Authors:** Sebastiano Andò, Ines Barone, Cinzia Giordano, Daniela Bonofiglio, Stefania Catalano

**Affiliations:** ^1^Department of Pharmacy, Health and Nutritional Sciences, University of Calabria, Rende, Italy; ^2^Centro Sanitario, University of Calabria, Rende, Italy

**Keywords:** breast cancer, leptin-signaling pathway, estrogen receptor, tumor microenvironment, EMT, stem cells

## Abstract

Adipokines represent likely candidates to mediate the increased breast cancer risk and the enhanced progression associated with obesity. Other contributors to obesity-related cancer progression are insulin/IGF-1 pathways and hormones. Among these, the adipokine leptin is the most intensively studied in both metabolism in general and in cancer due to the fact that leptin levels increase in proportion of fat mass. Leptin is primarily synthesized from adipocytes but it is also produced by other cells including fibroblasts. In this latter case, it has been well demonstrated how cancer-associated fibroblasts express leptin receptor and secrete leptin, which sustains a short autocrine loop and is able to target tumor epithelial cells enhancing breast cancer cell motility and invasiveness. In addition, it has been reported that leptin may induce breast cancer to undergo a transition from epithelial to spindle-like mesenchymal morphology, activating the signaling pathways devoted to the EMT. Thus, it emerges how leptin may play a crucial role in mediating malignant cell and tumor microenvironment interactions. Here, we present an overview of the role of leptin in breast cancer, covering the following topics: (1) leptin as an amplifier of estrogen signaling in tumor epithelial cells contributing to the promotion of carcinogenesis; (2) leptin as a crucial player in mediating tumor-stroma interaction and influencing EMT-linked mechanisms, that may sustain breast cancer growth and progression; (3) leptin and leptin receptor targeting as novel therapeutic strategies for breast cancer treatment.

## Introduction

Breast cancer, a complex and heterogeneous disease, is one of the most common human malignancies in women worldwide. Breast cancer development and progression depend on both the accumulation of various genetic alterations in the epithelial cells of the mammary gland, and the reciprocal interaction between tumor cell itself and its surrounding microenvironment (stroma). The stroma of the breast is composed of the extracellular matrix components (ECM) as well as several cellular types such as endothelial cells, pericytes, immune and inflammatory cells, adipocytes, and fibroblasts [termed cancer-associated fibroblasts (CAFs)] (1). In breast tumors, 80% of CAFs are in active form (2), and secrete high levels of various growth factors, cytokines, chemokines, and ECM degrading proteases (3) that by different mechanisms promote breast tumor onset and progression. In this regard, we recently identified leptin as a main regulator in the crosstalk between breast cancer cells and CAFs, adding, for the first time, leptin to the list of growth factors able to mediate tumor–stromal interaction (4).

Leptin, whose synthesis and plasma levels increase proportionally to total adipose-tissue mass (5, 6), is a pleiotropic molecule that regulates food intake, inflammation, immunity, cell differentiation, and proliferation of different cell types including cells of the breast (7). The activities of leptin are mediated through the transmembrane leptin receptor (ObR) encoded by db gene, a member of the class I cytokine receptor family that includes six isoforms different in the length of their intracellular tails (8). Leptin binding to ObR induces activation of multiple intracellular signaling such as Janus kinase 2-signal transducer and activator of transcription 3 (JAK2-STAT3), mitogen-activated protein kinase (MAPK), and phosphatidylinositol 3-kinase-protein kinase B (PI3K-AKT) pathways (9, 10) involved in different cellular activities. In the last decades, a plethora of data, strongly support the idea that leptin activity is correlated with breast cancer occurrence. Indeed, both leptin and its receptor are overexpressed in breast cancer, especially in higher grade tumors and are associated with distant metastasis (11, 12). Particularly, it has been extensively demonstrated using both *in vitro* and *in vivo* experimental models, that this adipokine modulates many aspects of breast cancer biology: e.g., increases cell proliferation and transformation, induces the expression of several cell cycle modulators, exerts anti-apoptotic effects, reduces efficacy of breast cancer treatment, influences cancer initiation processes (Table 1). Moreover, we and other authors have demonstrated that leptin can exert its activity also interacting with different signaling molecules.

**Table 1:** **Role of leptin in breast cancer growth and progression: *in vitro* and *in vivo* studies**.

Experimental models	Findings	References
***IN VITRO***
MCF-7	Increased cell proliferation	(4, 13–24)
T47D	
ZR75-1	
MDA-B-361	
SKBR3	
MDA-MB-231	
MCF-7	Increased cell transformation (anchorage-independent growth)	(4, 17, 25)
T47D	
SKBR3	
MCF-7	Up-regulation of cdk2, cyclin D1, E-cadherin, hyperphosphorylation of pRb, activation of estrogen receptor, induced expression of c-myc	(16, 17, 19, 21, 24, 26–28)
T47D	
ZR75-1	
MCF-7	Anti-apoptotic effect	(27, 29, 30)
ZR75-1	
MCF-7	Reduced efficacy of breast cancer treatment	(16, 25)
***IN VIVO***
Obese Zucker rats	Small percentage of carcinoma developed in obese compared with lean rats	(31)
MMTV/TGF-α Lep^ob/ob^	No transgene-induced mammary tumors development	(32)
MMTV/TGF-α Lep^db/db^	No transgene-induced mammary tumors development	(33)
MMTV/PyMT-α db/db^NSE/NSE^	Reduced mammary tumor growth and progression	(34)
	Enhanced mitochondrial	
	β-oxidation	
MMTV/Wnt1 tumors transplanted in Lep^ob/ob^	Suppressed mammary tumor growth and tumor-initiating cell survival	(35)

In this review, we will focus on the role of leptin in breast cancer highlighting the following topics: (1) leptin as an amplifier of estrogen signaling in tumor epithelial cells contributing to the promotion of carcinogenesis; (2) leptin as a crucial player in mediating tumor-stroma interaction and influencing EMT-linked mechanisms, that may sustain breast cancer growth and progression; (3) leptin and leptin receptor targeting as novel therapeutic strategies for breast cancer treatment.

## Functional Cross-Talk between Leptin and Estrogens

In addition to leptin, adipose tissue is a source of estrogens produced from androstenedione via aromatase conversion in postmenopausal women. Interestingly, several investigators have reported evidences that a functional cross-talk occurs between leptin and estrogen signaling network further contributes to breast carcinogenesis. Indeed, estrogen receptor (ER) α and ObRs are coexpressed in malignant mammary tissue and breast cancer cell lines (15, 17, 18) and it has been shown a positive association between serum leptin levels and elevated values of estrogen and progesterone receptor in patients with breast cancer (36, 37). Similarly, in human primary breast cancers, leptin receptor expression is positively correlated with tumor size and ER expression (38). Moreover, it has been reported that estradiol administration increases leptin mRNA expression in adipose tissue (39) and induces an enhanced leptin and ObR expression in MCF-7 breast cancer cells (40).

On the other hand, leptin is a potent modulator of the estrogen signaling pathway. Specifically, we reported that leptin is able to activate ERα transcriptionally through MAPK pathway in breast cancer cells in the absence of its natural ligand, reproducing the classic features of ERα transactivation, such as nuclear localization, down-regulation of its mRNA and protein levels, and up-regulation of classic estrogen-dependent genes (26). Recently, we have also demonstrated that a lysine to arginine mutation at residue 303 (K303R) within the hinge domain of ER may potentiate ERα’s role as an effector of leptin intracellular signal transduction, which may enhance cell proliferation, migration and invasiveness, contributing to the more aggressive phenotype of K303R-associated breast cancers (4). Moreover, leptin-induced activation of ERK and STAT3 has been correlated with an increased expression of ERα in breast cancer cells (41) and in breast tumors of nude-mouse xenograft model (42). This evidence is also confirmed by analysis of 33 patients with breast cancer at different stages of disease, demonstrating a significant association between the expression of leptin receptor and ERα (41).

Leptin also interferes with the estrogen antagonists ICI 182,780 and tamoxifen used in the treatment of ERα-positive tumors (16, 43).

Further evidence for the involvement of leptin in the paracrine stimulation of estrogen-responsive tissues is provided by its capacity to induce CYP19A1 synthesis in stromal cells isolated from the subcutaneous fat and breast adipose tissue of premenopausal women (44). In addition, Brown and colleagues have demonstrated that leptin stimulates aromatase expression in human breast adipose stromal cells through the regulation of LKB1/AMPK (AMP-activated protein kinase) pathway (45). Notably, they show that leptin downregulates LKB1 expression, accompanied by a decrease in AMPK phosphorylation, increased nuclear translocation of CRTC2 (CREB-regulated transcription coactivator 2), and a resulting increase in aromatase expression. Moreover, it has been reported that leptin enhances aromatase mRNA expression, protein content and its enzymatic activity in breast cancer cells, thereby promoting estradiol synthesis (13). This is mediated by AP1 (transcription activator protein 1) in ERK- and STAT3-dependent manner, since the presence of a MAPK inhibitor, ERK2- or STAT3-dominant negative constructs markedly attenuated the stimulatory effects of leptin on aromatase.

The enhanced local estrogen production induced by leptin could potentially also shape the breast cancer microenvironment. For example, local estrogens up-regulate TNF (tumor necrosis factor) receptor I expression in adipose-tissue fibroblast in an autocrine manner. Breast cancer epithelial cells produce large amounts of TNF, which through binding to TNF receptor I inhibits the differentiation of fibroblasts and preadipocytes into mature adipocytes, providing a molecular basis for the desmoplastic reaction commonly seen in breast cancer (46).

Overall, these studies indicate a biologically relevant cooperation between leptin and estrogen signaling pathways that might sustain the growth of estrogen-dependent breast cancer cells.

## Molecular Connection between Leptin and EMT

In patients with breast cancer, metastasis rather than the primary tumor is the main cause of death. Epithelial-to-mesenchymal transition (EMT), a normal physiological process for embryonic development and wound healing, is thought to be involved in cancer progression and metastasis. Indeed, EMT represents a critical step in which a normal polarized epithelial cell undergoes several biochemical changes to acquire a mesenchymal cell phenotype, such as acquisition of migratory and invasive capabilities and the loss of cell–cell adhesion and cell polarity, resulting in tumor aggressiveness, recurrence, and overall poor prognosis. Recently, it has been found that CAFs and fibroblasts from breast reduction specimens are able to induce EMT in breast cancer cell lines (47–49). The role played by CAFs in the development and progression of breast cancer relies on their ability to produce stromal ECM (extracellular matrix) proteins and secrete many growth factors and hormones, including insulin like growth factor (IGF)-I, IGF-II, epidermal growth factor (EGF), transforming growth factor (TGF)-α, TGF-β (3). For instance, stromal fibroblasts isolated from invasive breast cancer tissues promote aggressive phenotypes of breast cancer cells through EMT induced by paracrine TGF-β1 (50). Recently, we showed for the first time ObR RNA expression and leptin secretion in CAFs, proposing a novel integral role for leptin in mediating the bidirectional crosstalk between breast cancer cells and CAFs driving tumor growth and invasion (4). In parallel, it has been shown that leptin and its receptor initiate EMT via PI3K/Akt signaling pathway and β-catenin stabilization and nuclear translocation in breast cancer cells (51). The investigators observed in leptin-treated ERα-positive MCF-7 breast cancer cells as compared with untreated cells morphological phenotypic changes, including acquisition of fibroblast-like appearance, increased formation of pseudopodia from the cell membrane, reorganization of actin, and formation of stress fibers throughout cytoplasm. Such phenotypic differences were also reproduced upon leptin treatment in ERα-negative MDA-MB-231 and in MDA-MB-468 breast cancer cells. Exposure of cells to leptin stimulation resulted in loss of expression of E-cadherin and up-regulation of mesenchymal markers, which include N-cadherin, fibronectin, and vimentin. Expression of EMT inducers such as Snail, Slug, Zeb1, and Twist has also been found. The key mechanism underlying this important function of leptin involves a previously unrecognized interactions between leptin, metastasis-associated protein 1 (MTA1) and Wnt/β-catenin pathways.

Over the last decade, it is emerged that EMT is involved in the generation and function of breast cancer stem cells (BCSCs), a population of highly tumorigenic cells characterized by the expression of molecular markers (phenotype CD44^+^CD24^−^/ALDH^+^) that, in favorable microenvironments, self-renew, proliferate, and can differentiate to cells that include the bulk of the tumor mass. These cells appear to promote angiogenesis and escape immune surveillance, and chemo- and radio-therapy (52). Leptin has been reported to regulate and activate several signaling pathways and oncogenes, such as HER2, and AKT as well as transcription factors, such as STAT3 and NF-κB, which are critically implicated in BCSCs (53, 54). In addition, leptin also activates the Notch signaling pathway (55, 56) that is an important stem-cell signaling network. Interestingly, it has been found that leptin induces the expression of CD44 and ALDH1 in several cancer cell lines (53). Zheng et al. have reported a decreased tumor outgrowth and a functional depletion of BCSCs in obese leptin-deficient mice transplanted with murine mammary tumor virus (MMTV)-Wnt-1 tumor cells, showing that leptin signaling has an important role in tumor cell growth and stem-cell survival (35). Another study has also demonstrated that expression of Ob-R is a characteristic feature of CSCs, that display sensitized responses to leptin, such as STAT3 phosphorylation and activation along with Oct4 and Sox2 overexpression, thereby creating a self-reinforcing signaling network (57). More recently, it has been shown that the leptin receptor is necessary for maintaining CSC-like and metastatic properties in triple-negative breast cancer cells (58).

Given all the potential roles of leptin in the multistep processes of breast cancer progression, involving tumor initiation, primary tumor growth, invasion and metastasis, the leptin-signaling network is emerging as a novel therapeutic target for patients with breast cancers.

## Leptin as a Potential Target of the Novel Therapeutic Strategies for Breast Cancer Treatment

Several therapeutic approaches that could interfere with the actions of leptin and thereby prevent or delay leptin-related disease have been proposed (59).

### Soluble leptin receptors

Recombinant leptin-binding domains have been indicated as a treatment to block free leptin but they do not completely neutralize leptin activity due to a lower affinity for leptin compared to the intact soluble leptin receptor. (60, 61). However, several investigators have synthesized receptor-binding fragments able to inhibit leptin-induced proliferation and oncogenic signaling in both ER-positive and -negative breast cancer cells (62–64).

### Peptide-based leptin antagonists

An altered form of human leptin containing the mutation Arg128Gln was the first leptin antagonist synthesized. This mutation, tested in BAF/3 cells (an immortalized murine bone marrow-derived pro-B-cell line) stably transfected with the long form of human leptin receptor, exhibited binding properties similar to wild-type leptin, although it demonstrated reduced biological activity and weak antagonist properties when tested in cell proliferation assays (65). Another study has demonstrated that high doses of a short peptide sequences corresponding to amino acids 70–95 of human leptin is able to reverse leptin activity *in vitro* and *in vivo* (66–68). Moreover, Gertler et al. have demonstrated that mutations in sequence 39-42 (LDFI) of leptin lead to leptin muteins that still bind the receptor, but do not activate it (69, 70). Conversion of these amino acids to alanine resulted in the creation of leptin antagonists, being able to block endogenous leptin action in intact animals. Subsequent studies have (71–73) discovered another residue, Asp23, whose mutagenesis is associated with a dramatic increase in leptin-binding affinity. The simultaneous mutation of Asp23 with leucine and LDF with alanine, made it possible to synthesize the mutein D23L/L39A/D40A/F41A having a binding affinity receptor 60 times greater and antagonistic activity 14 times greater, *in vitro*, compared to the simple mutation LDF. Recently, we have synthesized a small peptide based on the wild-type sequence of leptin-binding site I, and demonstrated its efficacy in antagonizing leptin activity in breast cancer cell lines and in *in vivo* experimental models (data not published).

### Leptin-receptor-blocking antibodies

Leptin binding and signaling could be prevented by using high-affinity monoclonal antibodies that act as antagonists by interacting with the leptin receptor (74–76). A leptin-receptor-specific antibody has been tested in an *in vitro* study, and resulted in a reduction of both leptin signaling and leptin’s capability to activate monocytes and induce proliferation of peripheral blood mononuclear cells (75). The use of polyclonal antibodies that block leptin has also been proposed (74, 76). However, such antibodies could increase, rather than block, the activity of leptin *in vivo*, by prolonging its half-life in the circulation. The leptin-antibody complex would be larger than leptin alone, thereby decreasing kidney-mediated clearance of leptin (71). An alternative approach is represented by single-domain antibodies, i.e., small, monomeric antibody fragments of around 15 kDa that contain a single antigen-binding domain. These fragments are able to target the leptin receptor and block the ligand-induced conformational switch without interfering with leptin–leptin receptor interactions. They can bind to the leptin receptor with high affinity and do not cross the blood–brain barrier; thereby, they could selectively inhibit the peripheral activity of leptin (71).

## Conclusion

**Figure 1:**
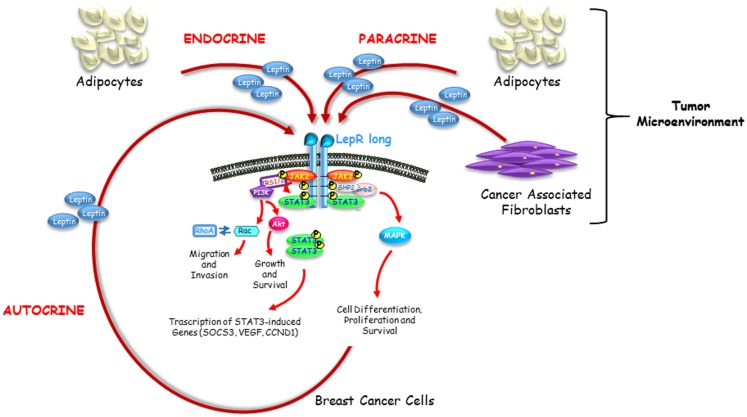
**Endocrine, paracrine and autocrine actions of leptin**. Leptin circulating at high levels in obesity impacts breast cancer initiation and progression in an endocrine manner. Leptin secreted by adipocytes and fibroblasts resident in breast tumor microenvironment acts on breast cancer cells in a paracrine fashion. Leptin produced by breast cancer cells supports tumor proliferation and invasion into the surrounding tissue through a short autocrine loop.

Adipokine leptin, produced by adipocytes, fibroblasts, and breast cancer cells, may act in an endocrine, paracrine as well as autocrine manner on breast cancer tissue (Figure 1). Activation of leptin-signaling results in concurrent activation of multiple oncogenic pathways leading to increased proliferation, acquisition of mesenchymal phenotype, enhanced migration, and invasion of tumor cells. Knowledge of the complex biological molecular network of leptin-signaling responsible for mammary carcinogenesis within tumor microenvironment provides a strong rationale for developing new agents, tailored to target the leptin and its receptor pathways, for therapeutic intervention in breast cancer treatment, particularly in women with obesity.

## Conflict of Interest Statement

The authors declare that the research was conducted in the absence of any commercial or financial relationships that could be construed as a potential conflict of interest.
